# Lifetime analysis of individual-atom contacts and crossover to geometric-shell structures in unstrained silver nanowires

**DOI:** 10.3762/bjnano.2.81

**Published:** 2011-11-03

**Authors:** Christian Obermair, Holger Kuhn, Thomas Schimmel

**Affiliations:** 1 Institute of Applied Physics and Center for Functional Nanostructures (CFN), University of Karlsruhe, Karlsruhe Institute of Technology (KIT), 76128 Karlsruhe, Germany; 2 Institute of Nanotechnology, Forschungszentrum Karlsruhe, Karlsruhe Institute of Technology (KIT), 76128 Karlsruhe, Germany

**Keywords:** nanowires, quantum point contacts, shell effect, silver

## Abstract

We study the crossover of quantum point contacts from (i) individual-atom contacts to (ii) electronic-shell effects and finally to (iii) geometric-shell effects in electrochemically deposited silver contacts. The method allows the fabrication of mechanically unstrained structures, which is a requirement for determining the individual atomic configuration by means of a detailed lifetime analysis of their conductance. Within the geometric-shell model, the sequence of conductance maxima is explained quantitatively based on the crystal structure data of silver, and the growth mechanism of the nanowires is discussed.

## Introduction

Recently, the first transistor on the atomic scale was demonstrated and generated much interest [1–5]. This atomic-scale transistor was formed by electrochemical deposition of silver into a nanoscale gap between two gold electrodes. Applying a control potential relative to a third, independent gate electrode allows opening and closing of an atomic-scale gap by the controlled and reversible relocation of individual atoms. In this way, switching between a quantized conducting “on-state” and an insulating “off-state” is performed. Even multilevel quantum switches on the atomic scale were demonstrated very recently [6]. The possibility of training special atomic configurations related to certain conductance values, and the high stability of the chosen conductance levels, are unique features of the electrochemical method [4–10]. Compared with mechanical setups, and separate from purely electrochemical methods, electromigration is another promising method to produce bistable contact configurations between integer quantum conductance values [11]. In order to effectively control the behavior of an electrochemically controlled atomic-scale transistor a detailed understanding of the mechanism of formation of the contacts bridging the nanoscale gap is necessary.

The conductance of nanocontacts strongly depends both on the type and number of atoms in the contact area and on the position of the atoms involved [12–13]. The conductance of single atoms was predicted theoretically [13–15] and investigated in detail by breaking thin wire junctions mechanically [16–19], exhibiting integer values of 1 *G*_0_ = 2*e*^2^/h for simple metals (such as alkali metals) as well as for gold and silver; the exact behavior depending on the signature of their chemical valence. Contacts with larger contact areas show a more complex behavior including electronic-shell effects and the filling of geometric shells. In both cases there are minima in the thermodynamic potential of the contact as a function of the radius, and radii with minima in their free energy are encountered more frequently during contact formation. In electronic shells these minima in free energy are related to the configuration of the electron system of the contacting atoms by analogy with the “magic” configurations in metal cluster. In geometric shells the free energy is lowered by the change of surface energy when completing a layer of atoms on the nanowire facets, which is also known from cluster physics [20–21]. Both the electronic- and the geometric-shell effect were intensely discussed for alkali metals [17,22] and later for noble metals [23] in mechanically fabricated atomic-scale contacts. However, it remained unclear how the results obtained with mechanically fabricated metallic point contacts are influenced by defects and distortions within the contacting area, generated during the mechanical fabrication process, and how the physics of shell effects and the structural fingerprints in conductance distributions are affected.

In contrast to the mechanical fabrication of contacts, the electrochemical method allows the fabrication of atomic-scale point contacts without the need to apply mechanical deformation. In this way, plastic deformations are avoided and highly stable and defect-free nanocontacts are produced [24–28]. This is especially true for silver; due to its high electrochemical exchange-current density, electrochemically deposited silver exhibits high mobility on its surface, allowing the fabrication of defect-free metallic point contacts [13]. A sufficiently high mobility of the atoms is needed to find stable configurations, corresponding to distinct shells, which, in turn, lead to clearly observable shell effects on the conductance.

Here, we study the transport properties and conductance-distribution statistics of electrochemically fabricated silver nanowires. We give a complete description of silver nanocontacts starting from individual atomic configurations (i.e., one or two atom contacts) proceeding to electronic-shell effects and finally accomplishing the crossover to the filling of complete geometric shells corresponding to crystallographic facets of the nanowire. A detailed lifetime analysis for selected contacts helps us to obtain a detailed understanding of the correlation between the physics of quantized electronic transport and the atomic structure of the nanocontacts.

## Results and Discussion

**Figure 1:**
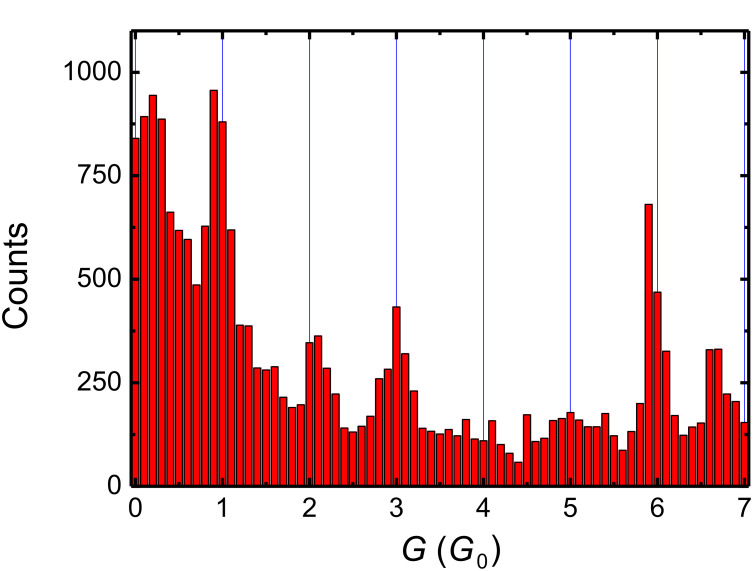
Conductance histogram of electrochemically deposited atomic-scale silver contacts giving evidence for different individual-atom and electronic-shell configurations. The histogram was obtained from >21000 conductance levels in the range between 0.01 *G*_0_ and 7 *G*_0_, each level being stable for longer than 200 ms as measured at room temperature, presented with a bin size of 0.1 *G*_0_ (1 *G*_0_ = 2*e*^2^/h). The histogram exhibits distinct maxima at defined integer multiples of *G*_0_ at 1 *G*_0_, 2 *G*_0_, 3 *G*_0_, 6 *G*_0_ and a less pronounced maximum at about 5 *G*_0_. Furthermore, there is a maximum at about 6.7 *G*_0_ which indicates the transition to the geometric-shell effect (Figure 4).

Figure 1 shows a conductance histogram for electrochemically deposited silver contacts obtained from 21385 conductance levels in the range between 0.01 *G*_0_ and 7 *G*_0_ with level each lasting longer than at least 200 ms. In this evaluation we counted the real number of conductance levels and not just the number of data points, as the latter (although often used in literature) would be misleading by overemphasizing the longer-living conductance levels. Consequently each data point in the histogram corresponds to a single complete conductance level. The histogram exhibits a sequence of distinct peaks at defined integer multiples of *G*_0_ at about 1 *G*_0_, 2 *G*_0_, 3 *G*_0_, 6 *G*_0_ and a less pronounced maximum, broader as compared to the others, at about 5 *G*_0_. The maximum at the noninteger conductance value at about 6.7 *G*_0_ indicates a different mechanism and is discussed below.

**Figure 2:**
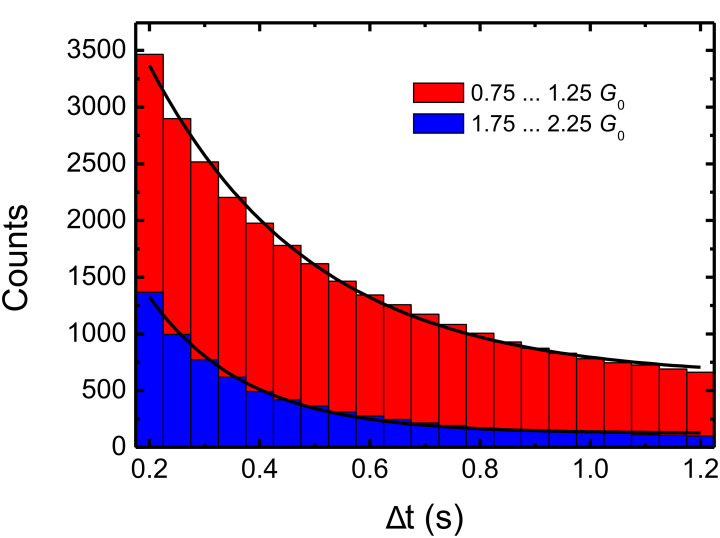
Number of conductance levels with level length greater than Δ*t* as a function of Δ*t*. The plot is given for two conductance levels: For the conductance range of 1 *G*_0_ ± 0.25 *G*_0_ and for the conductance range of 2 *G*_0_ ± 0.25 *G*_0_ taken from the histogram of Figure 1. By fitting an exponential decay function (solid lines) in the period between 0.2 s and 1.2 s, an average lifetime of the maximum at 1 *G*_0_ was estimated as τ_1_ ≈ 0.29 s and at 2 *G*_0_ as τ_2_ ≈ 0.18 s. Full results of the lifetimes analysis are found in Table 1.

The conductance value for a single-atom silver contact is expected to be 1 *G*_0_ [13,15]. A set of conductance peaks with the signature 1-3-(5)-6 can easily be associated with the values expected from a jellium model of electrons based on the degeneracy of transversal levels in a cylinder-symmetrical constriction [29–31], known as the “electronic shells” description. Even the less-pronounced maximum at approximately 5 *G*_0_ is conclusively explained by assuming slight deviations from perfect cylindrical contact geometries, the level 5 *G*_0_ being theoretically predicted to be less stable [30]. Only the appearance of a maximum corresponding to a conductance of 2 *G*_0_, which indicates a two-atom contact, is not compatible with this electronic-shell description. In light of this observation we further investigated the time behavior of the conductance levels in more detail. We analyzed the number of conductance levels within a fixed conductance range of distinct maxima, as a function of their minimum level length Δ*t*. Examples are given in Figure 2 for the maxima around 1 *G*_0_ and 2 *G*_0_. By fitting an exponential decay function in the period between 0.2 s and 1.2 s, an average lifetime τ was estimated as τ_1_ ≈ 0.29 s around the maximum at 1 *G*_0_ and τ_2_ ≈ 0.18 s at 2 *G*_0_. Selected average lifetimes of quantized conductance levels are listed in Table 1. These average lifetimes can be considered to be characteristic of the stability of the contacts. Table 1 shows the average lifetimes obtained for the conductance values corresponding to the electronic shells, with 0.29 s for the peak at 1 *G*_0_, 0.26 s for the peak at 3 *G*_0_ and 0.24 s for the peak at 6 *G*_0_. Strikingly, these values are up to 60% higher than the lifetimes obtained at the intermediate values, namely 0.18 s for the peak at 2 *G*_0_ and 0.19 s for the peak at 4 *G*_0_. These last two values are comparable to the lifetime of the background, or even slightly less stable than the background in between the maxima, here given for the example of 1.5 *G*_0_, which shows an average lifetime of τ = 0.21 s.

**Table 1:** Average lifetimes τ of contacts from different conductance ranges as derived from the data shown in Figure 2.

Conductance range	Average lifetime τ

1 *G*_0_ ± 0.25 *G*_0_	≈0.29 s
2 *G*_0_ ± 0.25 *G*_0_	≈0.18 s
3 *G*_0_ ± 0.25 *G*_0_	≈0.26 s
4 *G*_0_ ± 0.25 *G*_0_	≈0.19 s
5 *G*_0_ ± 0.25 *G*_0_	—^a^
6 *G*_0_ ± 0.25 *G*_0_	≈0.24 s
1.5 *G*_0_ ± 0.25 *G*_0_	≈0.21 s

^a^Due to the broadness of the maximum around 5 *G*_0_, τ could not be estimated.

**Figure 3:**
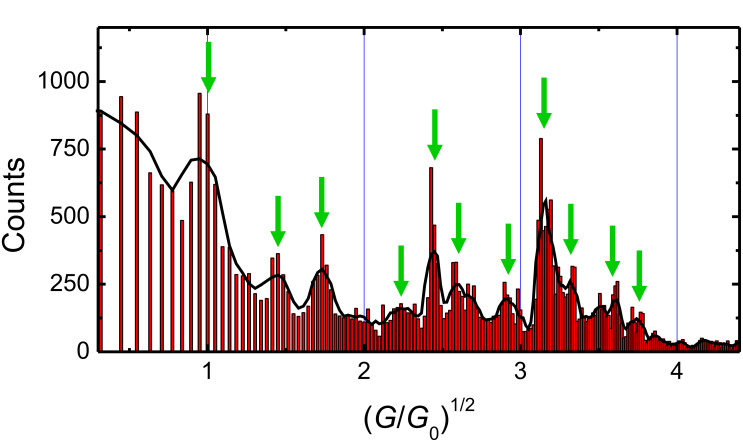
Conductance histogram for electrochemically deposited atomic-scale silver contacts giving evidence for the crossover from electronic-shells to geometric-shell configurations. The histogram was obtained from more than 36600 conductance levels in the range between 0.01 *G*_0_ and 19.9 *G*_0_, each longer than 200 ms. The *x*-axis is plotted as a function of the square root of conductance in units of *G*_0_ = 2*e*^2^/h, which is proportional to the contact radius *R* according to the Sharvin equation (see text). The solid line gives the two-neighbor average of the histogram data, which is used to identify the position of the maxima (marked by arrows).

**Figure 4:**
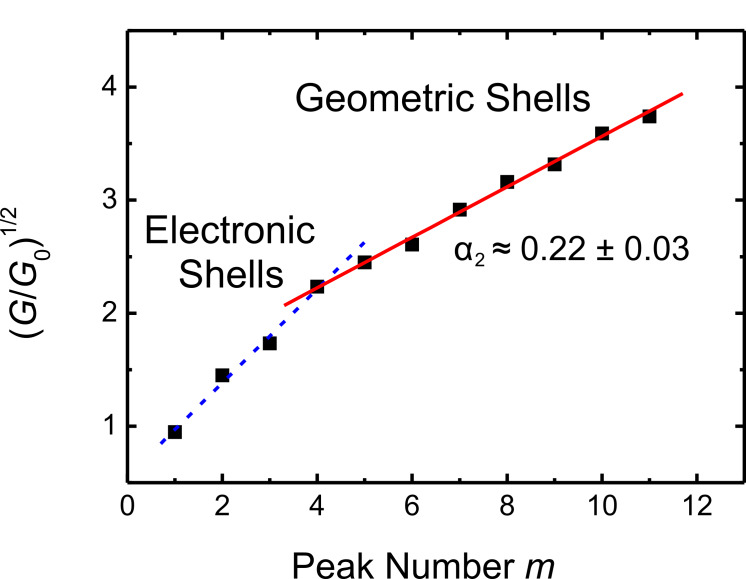
(*G*/*G*_0_)^1/2^ at the positions of the maxima observed in Figure 3 versus their sequentially numbered index. We observe two ranges: One up to the fourth maximum (dashed line) and another one from the fourth to the eleventh maximum with a slope of α_2_ ≈ 0.22 ± 0.03 (solid line). This behavior indicates two different mechanisms configuring the contact, the experimentally determined value of α_2_ being in excellent agreement with the value predicted by calculations.

**Figure 5:**
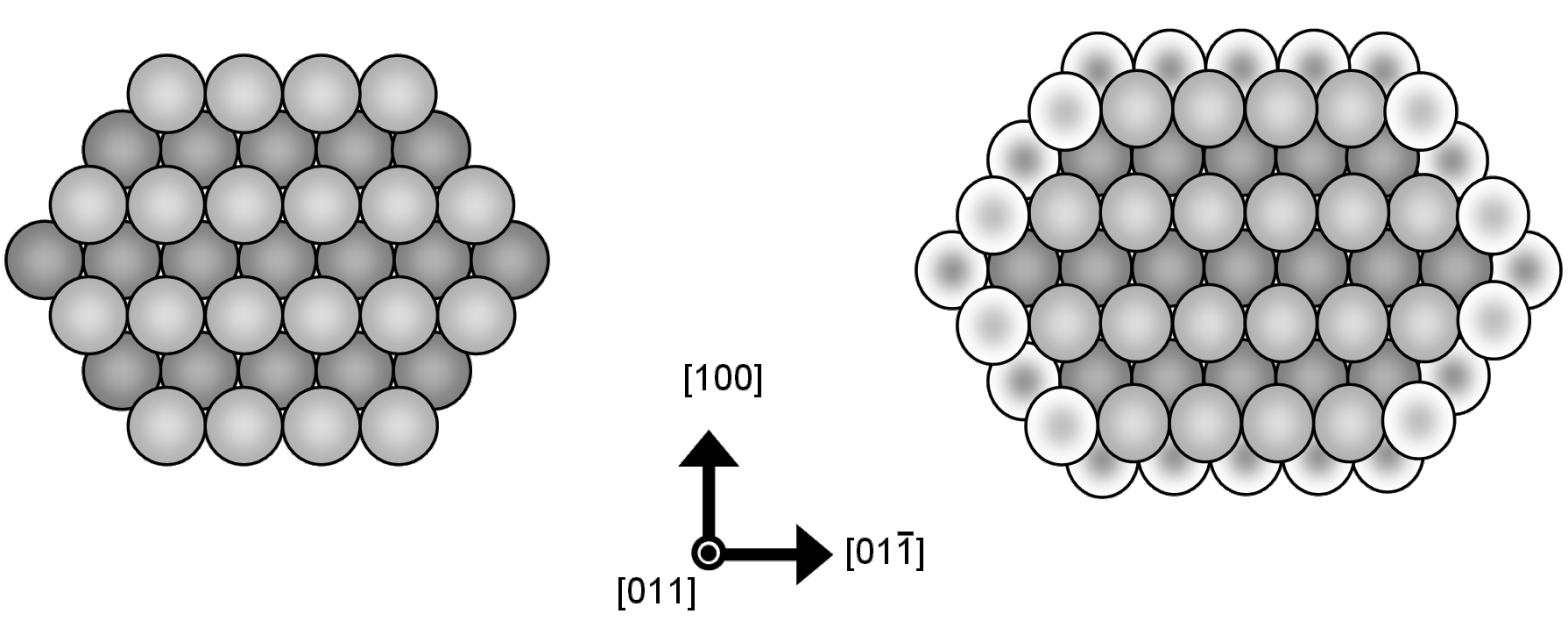
Illustration of a nanowire with fcc crystal structure and hexagonal cross-sectional area for two different diameters: On the right, one further shell is filled with atoms compared to the wire on the left.

An analysis of the entire observed conductance range up to 19.9 *G*_0_ obtained from 36608 conductance levels, each longer than 200 ms is given in Figure 3. As suggested by Yanson et al. [22], the number of observed conductance levels is plotted as a function of the square root of the conductance *G* in units of *G*_0_ = 2*e*^2^/h. The square root of *G*/*G*_0_ is proportional to the contact radius *R* according to the semiclassical Sharvin equation. The diagram can be divided into two ranges: The first range with (*G*/*G*_0_)^1/2^ ≤ 2.23 corresponds to the electronic shell sequence of maxima as discussed in relation to Figure 1. The sequence of maxima in the range with higher conductance (or contact radii) of (*G*/*G*_0_)^1/2^ > 2.23 show the striking feature that they are equidistant on the (*G*/*G*_0_)^1/2^ axis. This can be seen in more detail in Figure 4 were (*G*/*G*_0_)^1/2^ values at the positions of the maxima in Figure 3 are plotted versus their sequentially numbered index. Again, the range from the first to the fourth maximum can be explained by the electronic-shell structure. The second range from the fourth to the eleventh maximum exhibits an almost linear behavior with a slope of α_2_ ≈ 0.22 ± 0.03, indicating a different origin of the conductance quantization. Due to the proportionality of (*G*/*G*_0_)^1/2^ to the contact radius, this equidistant sequence corresponds to an increase in equidistant steps in the contact radius of the nanowires. This, in turn, can be explained by a subsequent filling of geometric shells with atoms around the contacting nanowire, also called the geometric-shell effect, as illustrated in Figure 5.

It can be expected that with increasing conductance, and consequently with increasing radius, the structure of the nanowires more and more tends to their crystallographic bulk structure. For silver this is an fcc structure with a hexagonal cross-sectional area, which was also found in high-resolution transmission electron microscopy studies of atomically thin silver nanowires [32]. In Figure 5 an illustration of such nanowires is given for two different diameters. The wires are directed along the 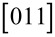 direction with six facets perpendicular to the 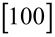, 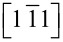, 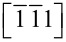, 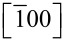, 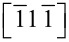 and 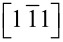 directions. Compared to the wire on the left in Figure 5, a further shell of atoms is added to the wire on the right. Within this structural model we can correlate each closed geometric-shell configuration and its corresponding cross-sectional area with a conductance according to the Sharvin equation. This gives a slope α for the trend of (*G*/*G*_0_)^1/2^ as a function of the peak index *m*:

[1]
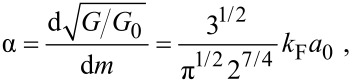


where *a*_0_ is the lattice constant of the cubic lattice and *k*_F_ is the Fermi wave vector. For a free-electron metal with fcc lattice we have *k*_F _*∙a*_0_* =* (12π^2^)^1/2^. From Equation 1 we obtain α ≈ 1.427. This value was calculated for the complete filling of one further shell of atoms. Filling a complete shell of a crystal wire of six-fold symmetry corresponds to the subsequent filling of six crystal facets. Assuming that each completely filled facet corresponds to an energy minimum and thus to a stable configuration, each filled facet will lead to a maximum in our diagram. Thus one would expect from the above calculation a slope of α_1/6_ = α/6 ≈ 0.238. This predicted value is in excellent agreement with the slope of α_2_ ≈ 0.22 ± 0.03 determined from our experimental values in Figure 4.

Thus, from the positions of the maxima in the conductance histogram of Figure 3, we can conclude a crossover from electronic-shells to geometric-shell configurations of the electrochemically deposited atomic-scale silver contacts. This transition can be explained by two competing effects: On the one hand there are oscillations of the free energy of the electron system of the contact, the amplitude of the local energy minima decreasing as 1/*R* due to shell filling [33]. On the other hand there is an oscillation in the surface energy due to the filling of geometric shells, for which the amplitude is roughly constant in radius [33]. Both effects can be of similar importance within a certain range of contact sizes, while for larger radii (corresponding to higher conductance values) the energetic effect of geometric-shell filling dominates over the effect on the electronic-shell filling.

The concrete conductance of transitions (or the number of maxima that can be observed in the electronically or in the geometrically dominated shell range) depends on the metal as well as on the experimental parameters: For mechanical break junction experiments, Mares et al. [23] found a crossover from electronic to geometric shells in silver at room temperate, at about 15 *G*_0_ in UHV and at about 22 *G*_0_ under ambient conditions. The authors argued that under ambient conditions adsorbates *decrease* the atom mobility, resulting in an enhanced stability of small contacts. In our case, we have exactly the opposite situation: The electrochemical environment leads to strongly *enhanced* surface-atom mobility, leading to a decrease of the transition towards the region of smaller contacts. The enhanced surface mobility results in a high degree of order of the contact area. Together with the fact that the deposition occurs without external mechanical strain, this explains the observation that electrochemically deposited silver junctions exhibit a dominance of geometric-shell effects beginning at much lower conductance levels or contact radii than in corresponding experiments with mechanical break junctions, leading to a crossover as early as 6 *G*_0_, as compared to 15 *G*_0_ and 22 *G*_0_ [23] in the case of mechanical break junctions.

Calvo et al. [28] found indications of shell effects in electrochemically deposited Au contacts but reported unstable contacts in the region below 20 *G*_0_. Compared to their experiments, electrochemically deposited silver contacts appear to exhibit a higher stability than the reported electrochemically deposited Au contacts. This may be due to the high electrochemical exchange-current density of silver, as electrochemical exchange currents provide a means for structural reconfiguration and for the healing of atomic-scale defects within the contact area [13,26]. The high degree of order of our contacts results in a transition between electronic- and geometric-shell effects at an unprecedentedly low conductance level.

## Conclusion

In conclusion, the detailed experimental study of the conductance of unstrained silver point contacts obtained by electrochemical deposition allowed the direct observation of the fingerprints of atom-by-atom and subsequent layer-by-layer growth of the metallic point contacts. We gave a complete quantitative description of the different stages of nanowire growth: First, individual-atomic contacts are formed, corresponding to only one or two atoms in their cross-sectional area. With increasing contact radius the conductance is dominated by the building of electronic shells, and finally the bulk crystallization leads to the dominance of geometric shells. Our experimental data are in excellent agreement with the corresponding calculations for silver. A detailed lifetime analysis of individual conductance levels indicated an increased stability of single-atom contacts or contacts stabilized by electronic shells. The stabilization of atomic-scale point contacts, in turn, is a key prerequisite for atomic-scale quantum electronics such as used in atomic transistors.

## Experimental

Two gold electrodes (with a thickness of about 100 nm) were deposited on a glass substrate with a gap of the order of 100 nm separating the electrodes. The gold electrodes were coated with an insulating layer, except for a small region with less than 25 µm in diameter around the gap, in order to keep the electrochemical leakage currents low (below 0.1%).

This arrangement was exposed to electrolytes consisting of 1 mM AgNO_3_ (p.a., Merck) and 0.1 M HNO_3_ (suprapure, Merck) as aqueous solutions within an electrochemical cell. Keeping a fixed voltage bias of 12.9 mV between the two ends of the leads allowed for simultaneous measurements of the conductance of the point contact. The (quasi-)reference electrode as well as the counter electrode consisted of 0.25 mm diameter Ag wire (99.9985%). All experiments were performed at room temperature and with the electrolyte exposed to ambient air. The data shown were obtained by deposition of silver into the gap between the working electrodes and simultaneous measurement of the conductance between them. When the conductance reached the maximum measuring range of the setup at about 20 *G*_0_, the deposition potential (about −10 mV versus Ag/Ag^+^) was changed to a dissolution potential (about 40 mV versus Ag/Ag^+^) until the gap opened again. Subsequently, a new deposition cycle was started automatically with the computer-controlled setup. The conductance curves were analyzed by using an algorithm that identifies sequences of consecutive conductance values within a given tolerance (here ±0.05 *G*_0_). Each identified level by definition had a minimum length of four consecutively measured data points (at 50 ms per data point). The conductance was recorded both during deposition and during dissolution of the contacts, and both processes were taken into account in our statistical analysis. However, in most cases, the opening process was much faster than the closing process, the complete opening process typically taking much less time than the minimum level length of 200 ms used for our evaluations. The data were derived from independent measurements of about 30 identical electrode setups. The data represent the collection of many different experiments obtained over a long time. By splitting the data into different sets each containing only part of the total data, comparable results were obtained, though with a somewhat reduced signal-to-noise ratio.
